# Classification system for Ai-enabled consumer-grade wearable technologies aiming to automatize decision-making about individualization of exercise procedures

**DOI:** 10.3389/fspor.2024.1500563

**Published:** 2025-01-29

**Authors:** Peter Düking, Sam Robertson, Hans-Christer Holmberg, Klaus-Hendrik Wolf, Billy Sperlich

**Affiliations:** ^1^ Department of Sports Science and Movement Pedagogy, Technische Universität Braunschweig, Braunschweig, Germany; ^2^ Institute for Health & Sport, Victoria University, Melbourne, VIC, Australia; ^3^ Division of Machine Elements, Luleå University of Technology, Luleå, Sweden; ^4^ Department of Physiology and Pharmacology, Biomedicum C5, Karolinska Institutet, Stockholm, Sweden; ^5^ Peter L. Reichertz Institute for Medical Informatics, TU Braunschweig and Hannover Medical School, Braunschweig, Germany; ^6^ Integrative & Experimental Exercise Science & Training, Department of Sport Science, University of Würzburg, Würzburg, Germany

**Keywords:** framework, innovation, mHealth, eHealth, Ai, technology, training signature

## 1 Introduction

Due to inter- and intra- individual variability in acute and chronic response at cellular and organ level (1, 2), individuals receive different health and performance benefits from exercise procedures (3–6). Individualization can be considered as one important aspect if optimal health and performance benefits should be achieved by exercise procedures. Decisions on individualization of aspects of exercise procedures (e.g., exercise intensity zones) can be performed e.g., based on data obtained by costly, one-time laboratory-based measurements performed by highly experienced personal. However, such measurements are available to a limited number of people with the necessary financial and time resources. To allow more individuals to gain access to individualized exercise procedures, there is a need for scientifically trustworthy, cost-effective, widely accessible technologies that even non-professionals can utilize which monitor, store, analyse and feedback data from individuals to inform decision making.

Due to technological advancements, miniaturization of hardware components such as e.g., battery, chipsets and sensor technologies allow for the cost-effective creation of wearable technologies which can monitor (currently with varying reliability and validity) an increasing number of parameters. Wearable technologies are among the top fitness trends as revealed by the American Colleague of Sports Medicine since years (7–10). Software developments allow the creation of advanced and artificial intelligence (Ai) algorithms which impact numerous aspects of our society (11, 12) including decision-making on individualization of exercise procedures (13).

Arguably, Ai may enable data processing and decision-making capabilities of more devices such as e.g., consumer-grade wearable technologies in the future. The hope is that the combination of consumer-grade wearable technologies which reliably and validly monitor and store individual data (e.g., heart rate, blood pressure, sleep related parameters) with explainable and trustworthy Ai algorithms which automatize the analyse of such data will improve and maybe even partly automatize decision-making regarding individualization of exercise procedures.

The current market for consumer-grade wearable technologies (which likely already use forms of Ai) is largely unregulated (14). Products are marketed by companies with the claim to support monitoring and automatization of decision-making to individualize aspects of exercise procedures (15, 16). However, available products differ in data reliability, validity, and data processing capabilities (17–19). While some products cannot monitor relevant parameters reliably and validly (17, 19), others process data automatically and provide guidance for individuals on how to individualize aspects of exercise. The variability in available Ai-enabled consumer-grade wearable technologies (Ai-WT) hinders comparison and selection for individuals seeking a product that supports or even automatize decision making regarding individualizing aspects of exercise procedures.

To overcome this problem, a system which helps to classify Ai-WT is warranted. Similar classification systems were e.g., designed for different levels of autonomous driving by the Society of Automotive Engineers (20) and are currently being discussed in the medical literature with respect to using artificial intelligence as an aid in making clinical decisions (21). For example, the Society of Automotive Engineers states five levels of autonomous driving, from driver assistance (level 1) such as lance centering to full driving automation everywhere and in all conditions (level 5) (20).

Hereinafter, we propose a classification system for consumer-grade wearable technologies which rely on forms of Ai to support or even automatize decision-making of individuals with respect to exercise procedures.

We believe such a classification system holds several benefits, including e.g., fostering discussions, clear communication using standardized vocabulary, as well as providing a basis for frameworks authorities to establish standards and regulations ensuring consumer-grade Ai-WT are developed, tested, and operated safely. Additionally, this classification system supports consumers to make informed decisions about selecting Ai-enabled consumer-grade wearable technologies regarding individualization of exercise procedures.

## 2 Theoretical background of the classification system—from data to decision

To create a classification system for Ai-enabled consumer-grade wearable technologies which aim to support decision-making of individuals with respect to exercise procedures, it is imperative to initially comprehend the way decisions can be derived from data. For this purpose, a model by Wali Van Lohuizen was developed already in 1986 (22) and recently introduced in the scientific literature for exercise and training procedures (23). This model includes six different classes, i.e., (1) Data, (2) Information, (3) Structured Information, (4) Insights, (5) Judgements, (6) Decision. Different operational steps have to be made to progress to the next class which are explained in detail elsewhere (24). It was argued that each subsequent class involves an increase in usability, i.e., lower classes have marginal use, whereas higher classes have more immediate use for decision-making with a simultaneous reduction in the risk for information overload (24).

**Figure 1:**
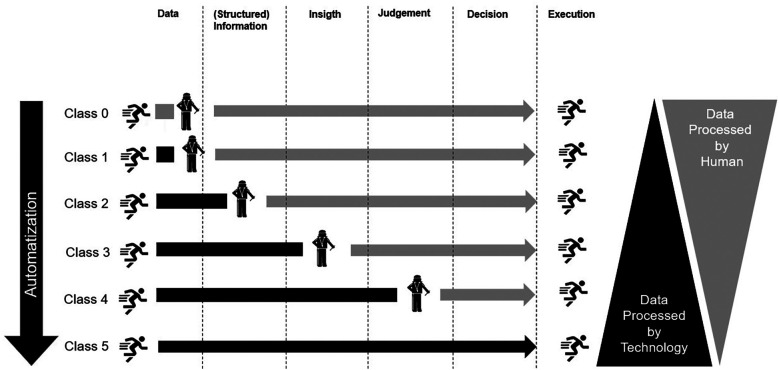
Schematic presentation of the proposed classification system for Ai-enabled consumer grade wearable technologies.

Based on this model, Ai-enabled consumer-grade wearable technologies aiming to assist decision-making procedures regarding individualization of exercise can be classified. As the class level increases, the technology processes, analyzes, and interprets more data, leading to greater automation and potentially reduced human involvement. To derive a functional, yet simple to use classification system, we combine classes 2 and 3 of the original model of Wali Van Lohuizen. Figure 1 depicts the classification system graphically.

## 3 Criteria to classify a technology

All criteria for lower classification must be fulfilled before higher classification can be considered, e.g., if any of the criteria for class 2 are violated, the Ai-WT cannot be placed in class 3.

### Class 0–no technology at all

In class 0, no Ai-WT is used. The decisions regarding exercise procedures are either based on (i) no data or (ii) data which is obtained through means unrelated to Ai-WT e.g., subjective human ratings.

### Class 1–automatization of data collection

To be assigned to Class 1, the Ai-WT must automatize data collection. Either data is automatically collected around the clock or in dedicated time periods. Dedicated time periods can be e.g., when the Ai-WT automatically detects time periods of interest (e.g., movement detection), or an individual activates data collection with minimal effort such as selecting a program on a smartwatch. Collected data needs to fulfill criteria for reliability and validity in the specific population and setting of its intended use. For this purpose, different recommendations are available, and we refer the reader to these articles (25–29).

Examples for Ai-WT assigned to this class include e.g., smartwatches which automatically detect certain exercise movements such as Burpees or Kettlebell thrusts (30).

### Class 2–automatization of data structuring

To be assigned in Class 2, Ai-WT must automatize structuring of the collected data. Depending on the specific Ai-WT, parameter and aim, this structuring can be e.g., chronologically in time (e.g., a time series of a heart rate measurement), accumulated instances (e.g., number of accumulated steps per day) and/or time synchronization of different parameters (e.g., synchronization of steps with heart rate and subjective parameters inserted manually into the Ai-WT system).

A potential example Ai-WT in this class could be a nowadays commonly available smartwatch collecting and structuring numbers of accumulated steps and heart rate over a course of a day or week.

### Class 3–automatization of insights

To be assigned in class 3, structured data needs to be analysed automatically, e.g., in relation to existing cut-off or threshold values to derive insights about the data. The cut-off or threshold values can be strictly defined based on current literature or determined individually by e.g., deviations from an individuaĺs “normal” baseline values. Examples for cut-off values derived from literature e.g., include the cut-off values for “normal” systolic and diastolic blood pressure established e.g., by the European Society of Cardiology and the European Society of Hypertension (31), and individually determined cut-offs include e.g., calculations of deviations from an individual baseline by more than one standard deviation (32). Ai-WT assigned to this class highlight that a parameter is within or outside of established thresholds, but do not derive a judgement about this. An user might use this insight about a parameter being out of range to elucidate the cause for this deviation and consequently might be able to derive a judgement and a decision to lead this parameter back into range.

For example, an Ai-WT might reveal that nightly assessed heart rate variability measures are out of range for an individual. Since there are different factors which affect nighty assessed heart rate variability, including e.g., food intake (33), training intensity (34), or room temperature variability (35), it is up to the user to determine the cause of the particular deviation of nightly assessed heart rate variability and to make decisions on how to act in order to bring heart rate variability back into the individual normal range.

### Class 4–automatization of judgements

To be assigned in Class 4, an Ai-WT need to provide a judgement for altering the exercise procedure based on data in terms of one or several recommendation(s). The final decision if or which of these recommendations is executed needs to be performed by an (experienced) individual. Recommendations need to be grounded on appropriate theoretical models derived from peer reviewed scientific literature (including population observational studies, non-randomized intervention studies and randomized controlled studies themselves or systematic reviews or meta-analyses thereof). This literature must confirm that the provided judgement does not cause harm and is more favourable to improve exercise procedures than a different judgement concerning an individual's exercise.

An Ai-WT could detect that an individual's nightly heart rate variability falls outside their usual range. It may also detect that the individual has increased their training compared to a previous period. Some evidence suggests that in certain athletic populations, when an individual's heart rate variability deviates from its usual range due to increased training load, a decrease in training load can enhance certain (sub-)maximal physiological variables compared to a predefined plan (32). Based on this, the Ai-WT may judge reducing the training load. However, the final decision should be made by an experienced individual, as other factors can influence HRV measurements. For example, an overload period might be intentionally planned, such as during a training camp.

### Class 5–automatization of decisions

To be assigned in Class 5, an Ai-WT need to make decisions about individualization of procedures. No experienced individual must confirm this decision. Appropriate peer-reviewed scientific literature (of the same nature as indicated above for Class 4) must show that the decision made by Ai-WT is relevant to the specific population and situation, does not cause harm to the health and/or performance of the individual; and improves the health and/or performance of the individual at least as much as a decision that a skilled human would make.

## 4 Practical utility, limitations and future perspective

The practical utility of the herein developed classification system for Ai-WT aiming to individualize aspects of exercise and training procedures include e.g., fostering discussions, clear communication using standardized vocabulary as well as providing a basis for frameworks authorities can use to establish standards and regulations ensuring consumer-grade wearable technologies are developed, tested, and operated safely. Additionally, this classification system empowers consumers to make informed decisions about selecting Ai-WT regarding individualization of exercise and training procedures.

Physiological adaptations evoked by exercise procedures are extremely complex and composed of multiple component subsystems (from molecular to systemic levels) which interact in non-linear, non-periodic and non-proportional ways which can hardly be analysed and interpreted in isolation (36). It requires an extensive knowledge of the innumerable causal connections that exist between the components of the system, a knowledge that is at least questionable to exist today (37). Increasing our understanding of these causal connections is crucial for decision-making to individualize exercise procedures, whether these decisions are made by humans or Ai-WT. Consequently, the major limitation connected with utilizing Ai-WT to assist or even automatize decisions for individualize exercise and training procedures may not be the technological possibilities *per se*, but rather the lack of scientific evidence of how to turn data into evidence-based decision. Therefore, it is also important not to exaggerate expectations about the role Ai-WT will ultimately play in evidence-based automatized decision-making regarding individualized exercise and training procedures. Drawing again on the analogy of self-driving cars from the introduction of this article, it is now generally accepted that fully safe autonomy under all circumstances (e.g., under difficult road conditions) will probably never be attained (38). Likewise, complete automatization and reliance on Ai-WT for evidence-based decision-making regarding individualizing exercise procedures, without human oversight and interpretation, seems unlikely (21) and ethically questionable.

## 5 Conclusions

Here, we propose a system of 5 levels for classifying Ai-WT for evidence-based automatized decision-making regarding individualized exercise and training procedures based on Van Lohuizen's model of decision-making. The classification system is beneficial for the standardization of Van Lohuizen's vocabulary for clear communication, provision of a foundation for regulatory frameworks to ensure appropriate development, testing, and operation of Ai-WT for exercise procedures.
